# Bone marrow mesenchymal stem cell-derived exosomes improve cancer drug delivery in human cell lines and a mouse osteosarcoma model

**DOI:** 10.3389/fonc.2024.1482087

**Published:** 2024-11-12

**Authors:** Wenquan Cai, Dawei He

**Affiliations:** Orthopaedics Department, Children’s Hospital of Chongqing Medical University, Chongqing Key Laboratory of Pediatrics, Ministry of Education Key Laboratory of Child Development and Disorders, National Clinical Research Center for Child Health and Disorders, China International Science and Technology Cooperation Base of Child Development and Critical Disorders, Chongqing, China

**Keywords:** bone marrow mesenchymal stem cell, exosomes, osteosarcoma, hybrid exosomes, doxorubicin

## Abstract

**Introduction:**

Osteosarcoma is the most common primary bone tumor. Patients require chemotherapy drugs with high-targeting ability and low off-target toxicity to improve their survival. Exosomes are biological vesicles that mediate long-distance communication between cells and naturally target their source sites. Exosomes derived from bone marrow mesenchymal stem cells (BMSCs) naturally target bone tumor sites, suggesting their potential as effective anti-tumor therapy vectors. In this study, we evaluated the potential of BMSC-derived exosomes in targeting osteosarcoma and serving as a carrier for doxorubicin (DOX).

**Methods:**

We isolated exosomes from human BMSCs and synthesized hybrid exosomes (HEs) by fusing these exosomes with liposomes. These HEs were loaded with DOX to produce a novel drug, HE/DOX.

**Results:**

We confirmed the successful synthesis of HE/DOX using fluorescence spectroscopy and estimated its size to be 151.1 ± 10.2 nm. HEs expressed the known exosomal proteins ALIX, CD63, and TSG101. Under acidic conditions similar to those observed in the tumor microenvironment, the drug release from HE/DOX was enhanced. In osteosarcoma cell lines and in a mouse osteosarcoma model, HE/DOX exhibited stronger tumor-inhibitory effects than free DOX.

**Conclusions:**

Our study demonstrates that BMSC-derived exosomes could effectively target osteosarcoma. Furthermore, HEs can serve as effective carriers of DOX, enabling the treatment of osteosarcoma. These findings highlight a promising direction for tumor-targeted therapy.

## 1 Introduction

Osteosarcoma is the most common bone tumor in children and adolescents and seriously endangers human health. Chemotherapy is an important treatment option that significantly improves the survival of patients with osteosarcoma; however, no progress has been made in osteosarcoma treatment in recent decades. Depending on tumor location and characteristics, reaching the site of its occurrence may be difficult for chemotherapy drugs, and this reduces their efficacy (1–4). Therefore, developing new drugs or carriers is necessary to improve treatment strategies and survival of patients with osteosarcoma.

Extracellular vesicles are nanoparticles secreted by most cells and can be divided into three types based on their biogenesis: microvesicles, exosomes, and apoptotic bodies (5). Exosomes are the most common extracellular vesicles, with a size of approximately 30–150 nm, and can load cellular contents, such as proteins, lipids, and nucleic acids, during their biogenesis (6). Exosomes, which have a phospholipid bilayer similar to typical drug-delivery liposomes, have been widely studied as drug delivery carriers. Drug delivery by exosomes can increase blood circulation time, improve drug stability, and allow accumulation in target tissues (7, 8). The advantages of using exosomes as drug delivery carriers are reflected in their ability to evade immune rejection. Although artificial lipid-based nanocarriers have shown significant progress in targeting, immune rejection still occurs (9). Natural exosomes exist in organisms and can be secreted by various cells, such as red blood cells, white blood cells, macrophages, and stem cells. Exosomes derived from their own sources can have good biocompatibility and low immunogenicity in organisms and can escape capture by the immune system (10–12).

Exosomes are secreted by various cells. Stem cells are ideal drug carriers for cell production as they secrete more exosomes, are easy to obtain from the human body, can be expanded *in vitro*, and have natural tumor targeting and low immunogenicity (13–15). Exosomes also possess natural homing abilities to target their source sites (16). For example, bone marrow mesenchymal stem cell (BMSC)-derived exosomes can actively home into the site of osteosarcoma to achieve targeted tumor delivery and are, therefore, suitable as carriers for drug loading (17, 18).

Although exosome production by stem cells is high, it still cannot meet the treatment needs. Moreover, owing to the influence of separation technology, exosomes as drug carriers require considerable time, financial resources, and professional technicians (19). To address the demand for exosomes as drug carriers, studies have explored membrane fusion technologies, including freeze-thaw and incubation methods (20–22). Membrane fusion technology synthesizes nanoparticles less than 200 nm in size by hybridizing exosomes with liposomes to generate hybrid exosomes (HEs). These HEs have the advantages of both exosomes and liposomes. Exosomes have many advantages as natural drug delivery systems, but their production limits their application; HEs make up for this shortcoming as they can be produced on a large scale. In addition, HEs can overcome the immune rejection and toxicity associated with liposomes. Recently, macrophage-derived exosomes hybridized with liposomes were used to successfully deliver chemotherapeutic drugs for tumor treatment (23).

We hypothesized that owing to their homing properties BMSC-derived exosomes could target osteosarcoma. To test this hypothesis, in this study, we examined the distribution of BMSC-derived exosomes *in vivo*. In addition, we hybridized exosomes with synthetic liposomes to prepare HEs and transfer doxorubicin (DOX) to osteosarcoma cells. This drug delivery system offers an effective method for treating osteosarcoma (24). It is important to note that in this study, we used small extracellular vesicles derived from BMSCs, with a diameter <200 nm, and the term “exosome” in this article refers to such small extracellular vesicles.

## 2 Results

### 2.1 Isolation and characterization of HEs loaded with DOX

**Figure 1:**
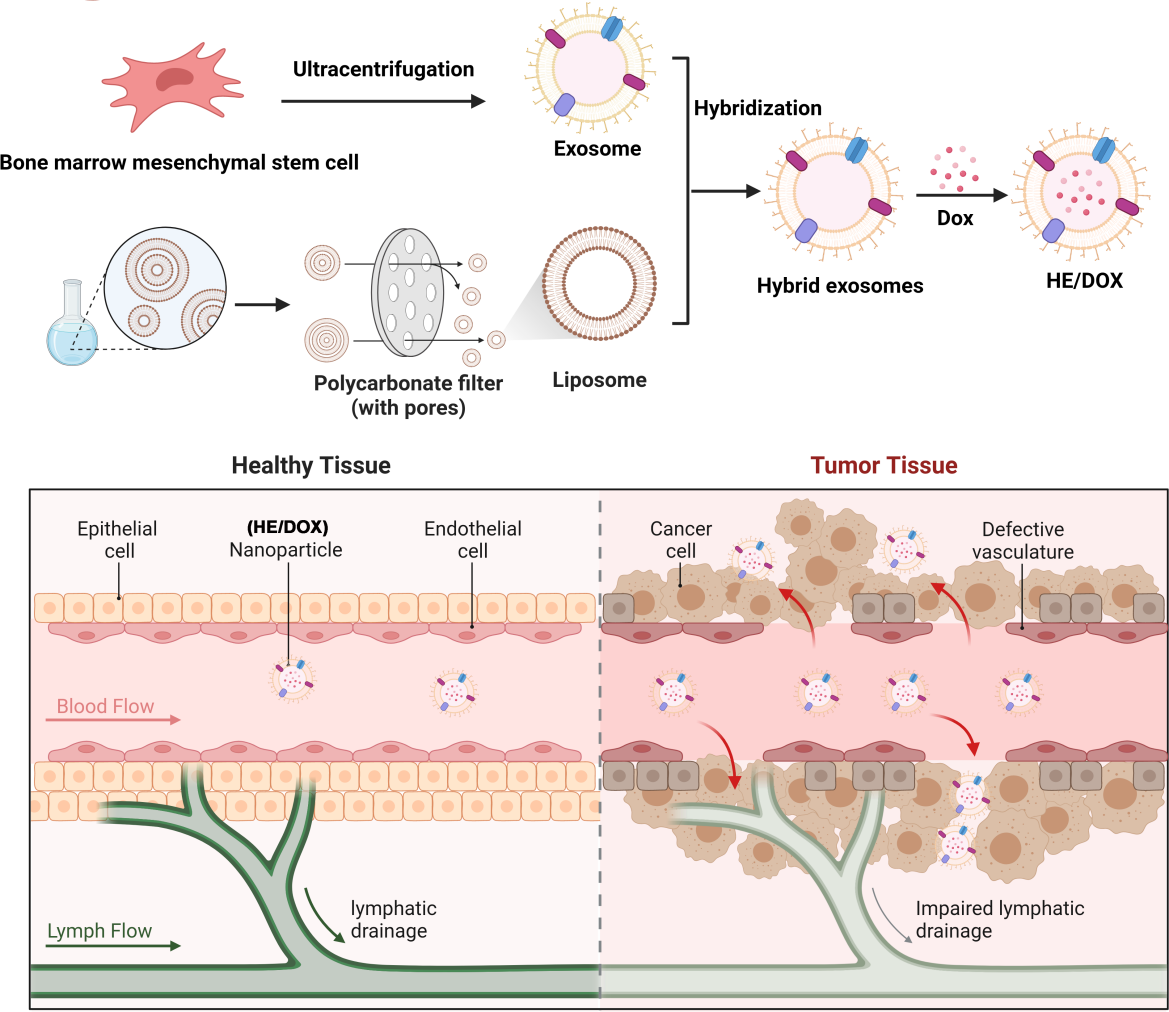
Schematic diagram of a drug delivery system involving the fusion of BMSC-derived exosomes and liposomes to generate HEs, which were loaded with DOX for anti-osteosarcoma therapy. The upper panel illustrates the process of HE/DOX preparation. The lower panel shows that HE/DOX easily penetrates the tumor tissue through vascular endothelial cells, achieving effective DOX delivery.

**Figure 2:**
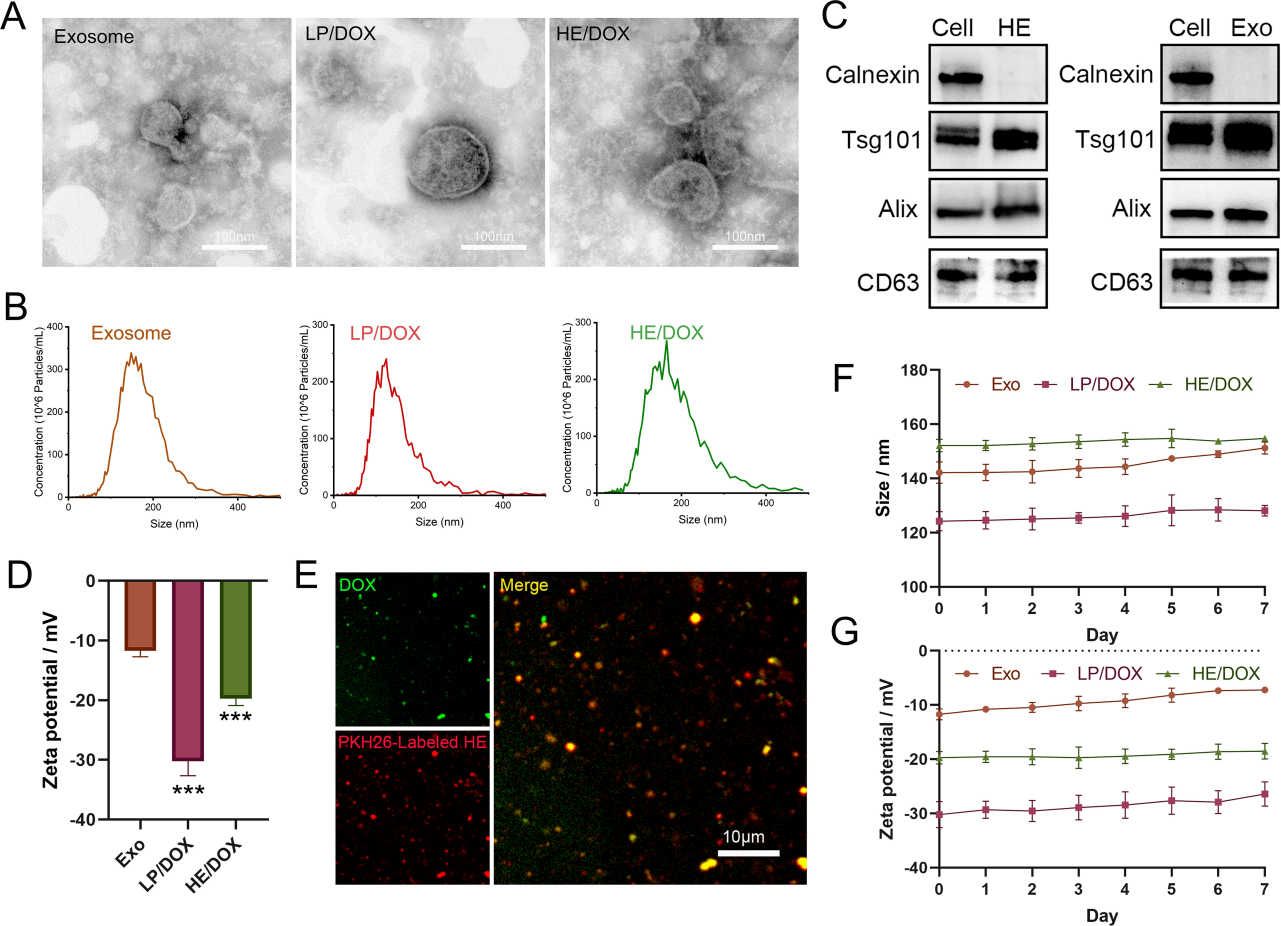
Morphological identification and protein characteristics of exosomes, LP/DOX, and HE/DOX. **(A)** Transmission electron microscopy images showing the morphology of exosomes, LP/DOX, and HE/DOX. **(B)** Nanoparticle size tracking analysis revealed the size distribution and concentration of exosomes, LP/DOX, and HE/DOX. **(C)** Western blot analysis of exosomal markers (ALIX, TSG101, and CD63) and a negative marker (calnexin) on HEs and exosomes (Exo). **(D)** Zeta potential of exosomes, LP/DOX, and HE/DOX. **(E)** Co-localization analysis and confocal images of pkh26-labeled HEs (red) and DOX (green). **(F)** Changes in the size of exosomes (Exo), LP/DOX, and HE/DOX under storage conditions at 4°C. **(G)** Changes in the zeta potential of exosomes (Exo), LP/DOX, and HE/DOX under storage conditions at 4°C. *** *P* < 0.001. All the statistical graphs are the results of three experiments repeated, and the pictures are the representative results of the three experiments.

First, we extracted exosomes secreted by BMSCs to prepare HEs by fusing them with liposome membranes and then prepared HEs loaded with DOX (HE/DOX) and liposomes loaded with DOX (LP/DOX) using the ammonium sulfate gradient method (**Figure 1**). Transmission electron microscopy revealed the morphology of the exosomes, HE/DOX, and LP/DOX, all of which showed a double-layer membrane structure (**Figure 2A**). Nanoparticle tracking analysis demonstrated that the peak diameter of HE/DOX was 151.1 ± 10.2 nm (**Figure 2B**), similar in size to exosomes and LP/DOX. Western blot analysis showed that HEs expressed the exosomal markers ALG-2-interacting protein X (ALIX), CD63, and tumor susceptibility 101 (TSG101) but did not express the negative marker calnexin (**Figure 2C**), indicating the successful fusion of liposomes and exosomes. In addition, we measured the surface charge of the exosomes, LP/DOX, and HE/DOX, which had zeta potentials of −11.7 ± 0.8, −30.2 ± 2.0, and −19.7 ± 0.9 mV, respectively. The difference between LP/DOX and HE/DOX was statistically significant when compared to the exosome group (exosome *vs.* LP/DOX, *P* = 0.0003; exosome *vs.* HE/DOX, *P* = 0.0008) (**Figure 2D**). We used PKH26 to label HEs (red), and fluorescence overlap was observed using the intrinsic fluorescence of DOX (green) under confocal microscopy, which demonstrated that DOX was encapsulated within HEs (**Figure 2E**). To test the stability of the exosomes, LP/DOX, and HE/DOX, they were stored at 4°C and their stability in terms of particle size and zeta potential was measured. Compared with the zeta potential on the first day, the average changes in zeta potential in the exosome, LP/DOX, and HE/DOX groups on the last day were 4.5 ± 0.6, 3.8 ± 0.3, and 1.2 ± 0.5 mV, respectively. The differences between the groups were statistically significant (*P* = 0.0004). The results showed that HE/DOX was more stable than the exosomes (**Figures 2F, G**).

### 2.2 DOX loading and release study

**Figure 3:**
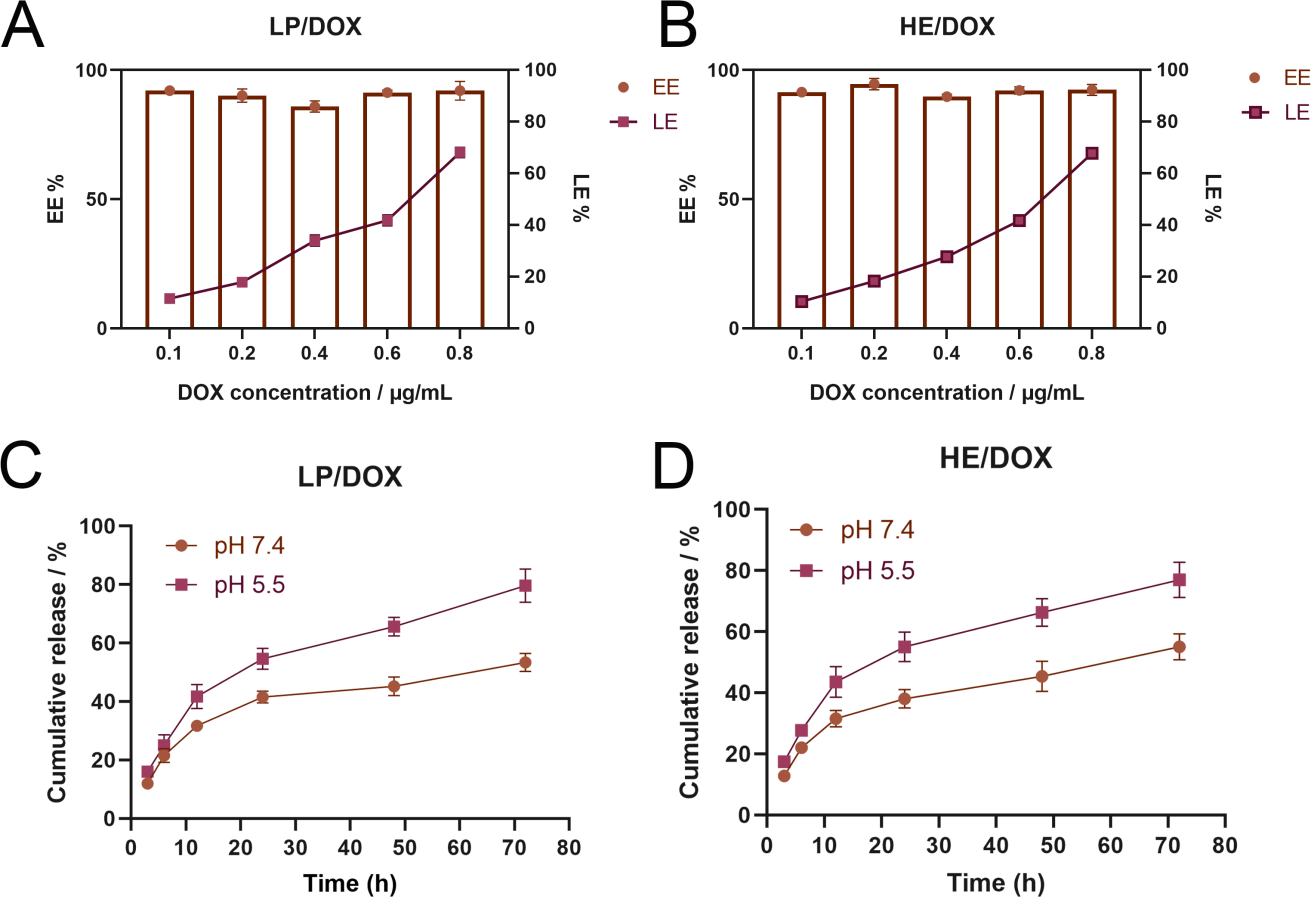
Loading and release of DOX by LP/DOX and HE/DOX. **(A)** Encapsulation efficiency (EE) and loading efficiency (LE) of DOX by LP/DOX. **(B)** EE and LE of DOX by HE/DOX. **(C)** Drug release efficiency of LP/DOX under pH 5.5 and 7.4. **(D)** Drug release efficiency of HE/DOX under pH 5.5 and 7.4. All the statistical graphs are the results of three experiments repeated, and the pictures are the representative results of the three experiments.

We studied the drug loading and release profiles of HE/DOX. The results showed that even at a DOX concentration of 0.8 mg/mL, the drug encapsulation efficiency was high (approximately 90%), whereas the loading efficiency continued to increase and could reach approximately 70% (**Figure 3B**). LP/DOX had a drug-loading profile similar to that of HE/DOX (**Figure 3A**), indicating that the extracellular vesicle membrane did not change the drug-loading properties of the liposomes. Leakage rates of HE/DOX and LP/DOX were also measured. **Supplementary Figure S1** shows that at 4°C, no significant leakage of HE/DOX and LP/DOX was observed within 7 d, indicating that the HE/DOX membrane was highly stable.

In addition, we studied the drug release profiles of HE/DOX and LP/DOX under simulated physiological conditions (pH 7.4) and in a tumor endolysosomal compartment interior environment (pH 5.5). As shown in **Figure 3**, HE/DOX and LP/DOX were rapidly released in the acidic environment; however, their release was slower under physiological conditions (**Figures 3C, D**).

### 2.3 BMSC-derived exosomes home into osteosarcoma cells *in vitro*

**Figure 4:**
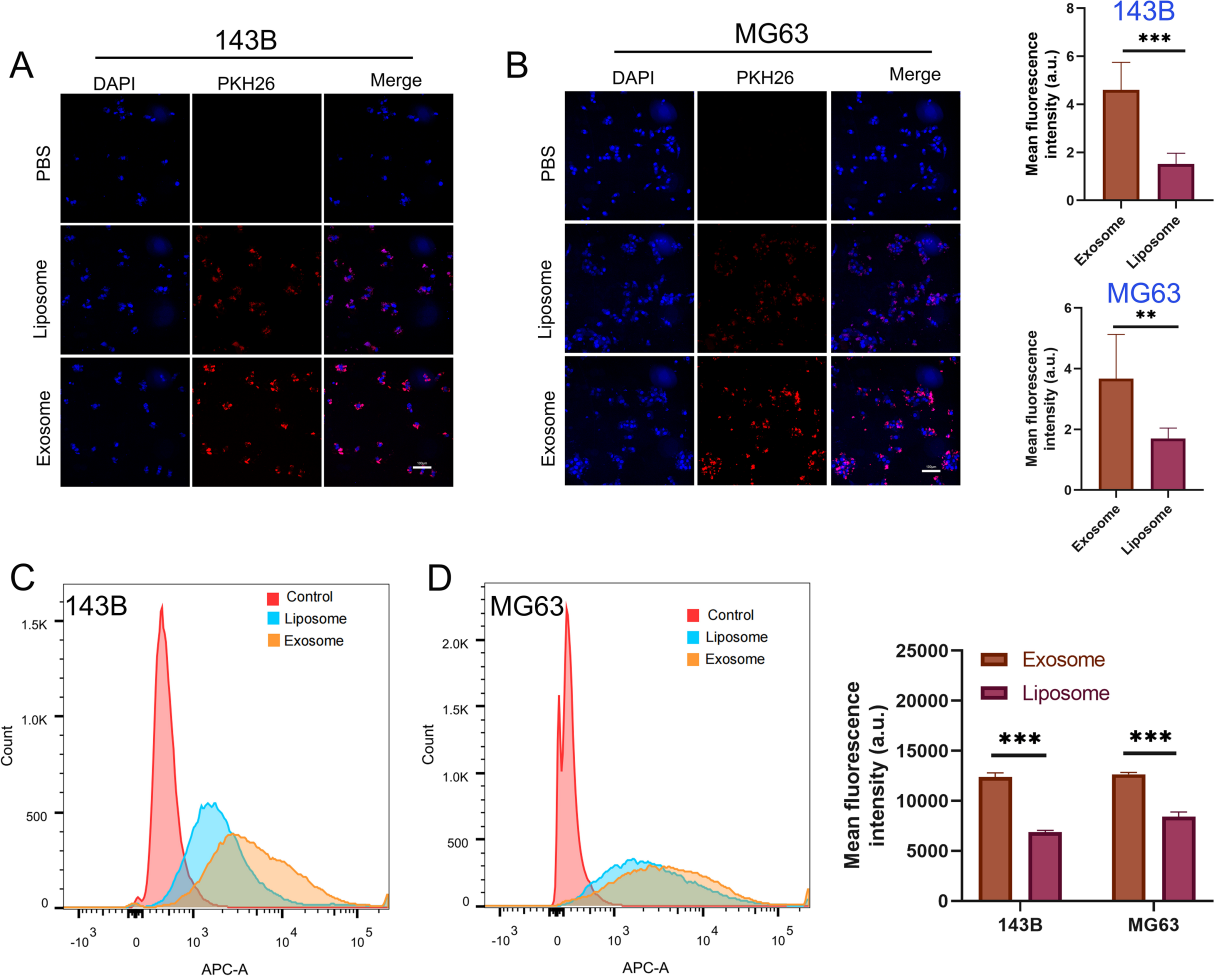
Cell uptake and internalization of exosomes and liposomes. **(A, B)** Quantitative representative confocal images and cellular uptake of exosomes and liposomes by 143B and MG63 osteosarcoma cells, Red represents PKH26-labeled exosomes and liposomes, and blue represents DAPI-labeled nuclei. **(C, D)** Quantification of osteosarcoma cell uptake of exosomes and liposomes using flow cytometry. **P < 0.01, ***P < 0.001. All the statistical graphs are the results of three experiments repeated, and the pictures are the representative results of the three experiments.

To determine whether BMSC-derived exosomes enter osteosarcoma cells *in vitro*, we isolated exosomes from BMSCs and used liposomes as a control. PKH26-labeled BMSC-derived exosomes and liposomes were co-cultured with the osteosarcoma cell lines 143B and MG63 for 12 h, and fluorescence was quantified using confocal microscopy. The results showed that the uptake of BMSC-derived exosomes by 143B and MG63 cells was significantly higher than that of liposomes (**Figures 4A, B**). In addition, we co-cultured DiD-labeled exosomes with osteosarcoma cells and used flow cytometry to quantify exosome uptake by detecting fluorescence. The results also indicated that BMSC-derived exosomes were taken up by osteosarcoma cells more easily than were liposomes (**Figures 4C, D**).

### 2.4 Tumor uptake and inhibition

**Figure 5:**
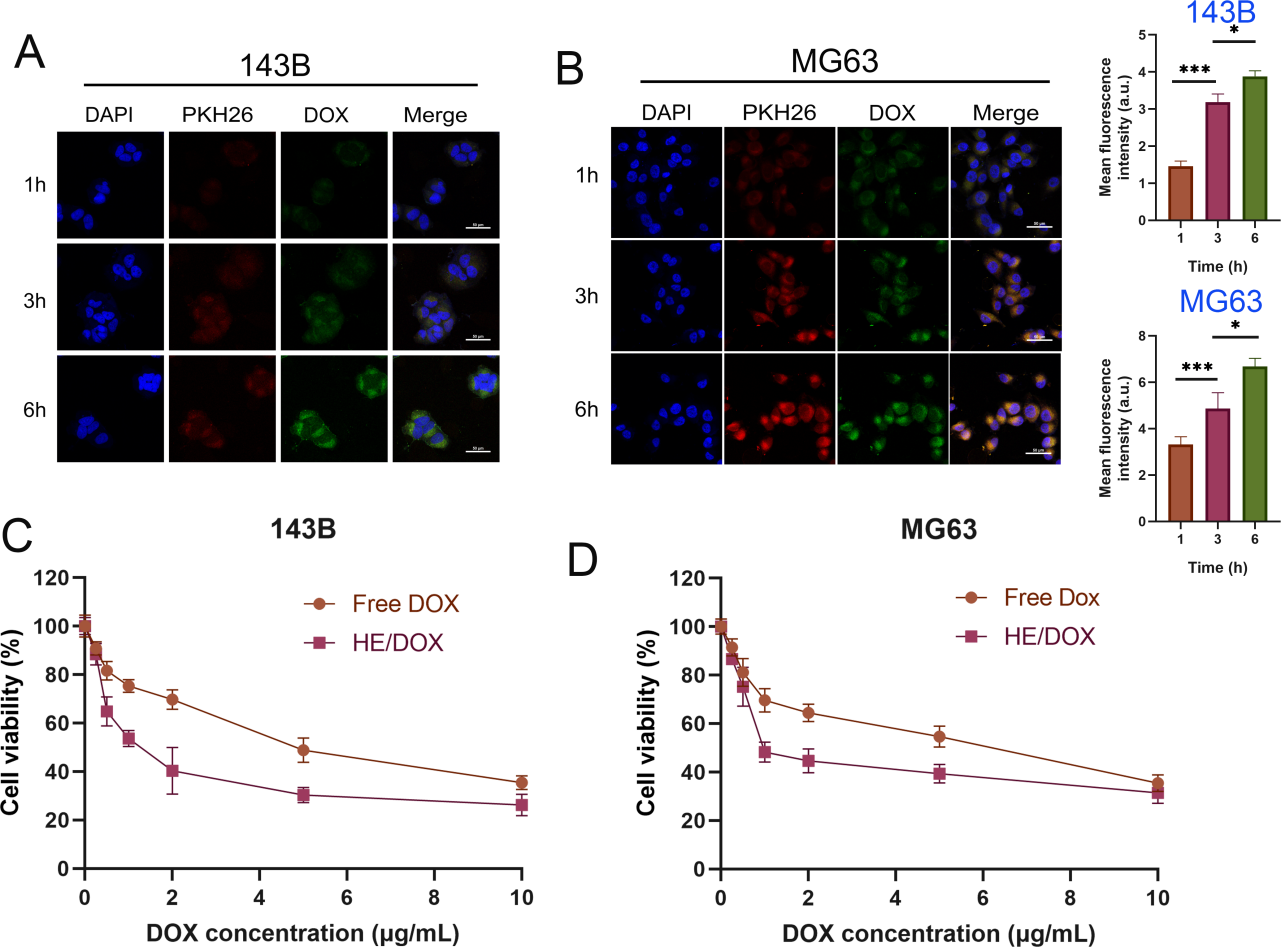
Cellular uptake and cytotoxicity of HE/DOX. **(A, B)** Quantitative representative confocal images and cellular uptake of HE/DOX. Red represents PKH26-labeled HEs, green represents DOX, and blue represents DAPI-labeled nuclei. **(C, D)** Cell viability following administration of free DOX and HE/DOX of the osteosarcoma cell lines 143B and MG63. *P < 0.05, ***P < 0.001. All the statistical graphs are the results of three experiments repeated, and the pictures are the representative results of the three experiments.

To investigate the uptake of HE/DOX by tumor cells, we co-cultured PKH26-labeled HE/DOX with 143B and MG63. To study the temporal pattern of osteosarcoma cell internalization of HE/DOX, we observed internalization at 1, 3, and 6 h using confocal microscopy (**Figures 5A, B**). Osteosarcoma cell internalization of HE/DOX was significantly time-dependent, showing a distinct increase in internalization over time.

We used the Cell-Counting Kit- 8 (CCK-8) assay to evaluate the growth inhibition/cytotoxic effects of free DOX and HE/DOX on 143B and MG63 cells (**Figures 5C, D**). The dose-response curves showed that HE/DOX had a significantly stronger inhibitory effect on 143B and MG63 cells than free DOX. The half-maximal inhibitory concentrations (IC_50_) of HE/DOX on 143B and MG63 cells were 1.5 and 1.97 μg/mL, respectively, which were significantly lower than the IC_50_ of free DOX at 4.81 and 4.89 μg/mL, respectively. These results indicate that HE/DOX has an inhibitory effect on osteosarcoma and enhances the toxicity of free DOX. Additionally, we investigated the cytotoxic effects of BMSC-derived exosomes, liposomes, and HEs on cells (**Supplementary Figure S2**). The dose-response curves indicated that BMSC-derived exosomes, liposomes, and HEs did not show significant cytotoxicity.

### 2.5 Therapeutic effects of HE/DOX *in vivo*

**Figure 6:**
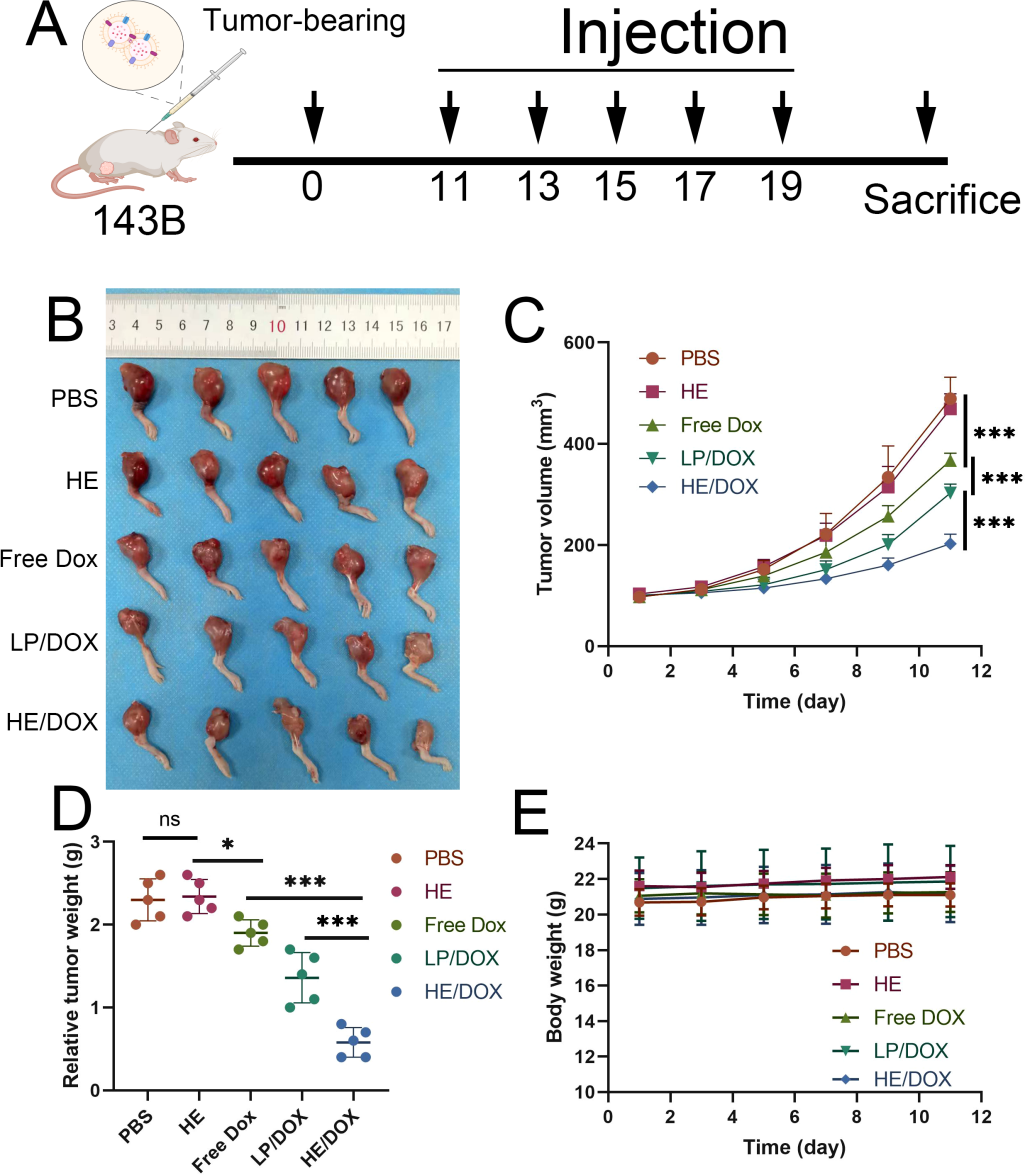
*In vivo* antitumor effect. **(A)** Schematic diagram for the treatment of a mouse orthotopic tumor-bearing model. **(B)** *In situ* images of osteosarcoma treated with PBS, HEs, free DOX, LP/DOX, or HE/DOX (*n* = 5). **(C)** Changes in tumor volume over time. **(D)** Tumor mass of mice at the endpoint. **(E)** Weight of mice following treatment. *P < 0.05, ***P < 0.001. All the statistical graphs are the results of three experiments repeated, and the pictures are the representative results of the three experiments.

To evaluate the inhibitory effect of HE/DOX on tumors, we injected phosphate-buffered saline (PBS), HEs, free DOX, LP/DOX, or HE/DOX into orthotopic tumor-bearing mice every other day for a total of five injections. **Figure 6A** shows the treatment of the nude mouse tumor model. The dose of DOX administered to each mouse in the treatment group was 5 mg/kg. The results showed that mice in the PBS and HE groups had the fastest increase in tumor volume, whereas those in the free DOX, LP/DOX, and HE/DOX groups showed slow tumor growth (**Figures 6B–D**). Compared to the free DOX group, the LP/DOX group showed better tumor growth inhibition. Furthermore, HE/DOX showed a more successful therapeutic effect than LP/DOX. Additionally, no significant difference in body weight was observed between the groups during treatment (**Figure 6E**), indicating that the drugs had no significant side effect on mice.

## 3 Discussion

Exosomes serve as carriers for drug delivery and as substances for intercellular communication. Exosomes from BMSCs contain specific molecular libraries, including proteins, lipids, DNA, and RNA that participate in the communication and cellular processes of osteosarcoma cells (25). Exosomes closely associated with osteosarcoma cells as nanovesicles serve as effective drug delivery carriers. Our findings demonstrate that BMSC-derived exosomes can target osteosarcoma cells, a targeting capability that can be utilized by fusing them with liposomes to effectively inhibit osteosarcoma growth with DOX. Specifically, we fused exosomes from BMSCs with liposomes to carry DOX, forming a composite nanodrug that transports chemotherapeutic drugs to the tumor site and inhibits tumor growth.

HEs formed by the fusion of exosomes and liposomes express similar marker proteins as exosomes. Therefore, HEs may have characteristics similar to those of exosomes; for example, BMSC-derived exosomes can actively home into osteosarcoma. We speculate that the ability of HEs to target osteosarcoma may be related to the targeting by exosomes.

Owing to their lack of natural components, synthetic materials exhibit strong immunogenicity, making them prone to clearance by the immune system. Therefore, surface modifications of nanomaterials can reduce their immunogenicity, enabling their effective delivery to tumor cells. Previous studies have reported that synthetic materials, with surface modifications using tumor cell membranes, can effectively target such type of tumor cells (26).

DOX, a key chemotherapeutic drug for osteosarcoma, has limited applications owing to its accumulation in non-targeted organs and particularly its off-target cardiac toxicity (27). Exosomes, acting as encapsulation carriers, can increase the solubility of certain natural compounds (28) and promote endocytic processes through expressed transmembrane or anchored proteins (29). Unlike liposomes, the integrin-related transmembrane proteins on the surface of exosomes can activate “don’t eat me” signals to protect loaded contents from immune system phagocytosis (30, 31). The results of confocal and flow cytometry analyses showed that BMSC-derived exosomes were taken up more readily by osteosarcoma cells than liposomes, which also suggests that liposomes hybridized with exosomes may be more advantageous as drug carriers for the treatment of osteosarcoma than liposomes alone. We believe that this is one of the reasons why HE-DOX has a stronger inhibitory effect on osteosarcoma *in vivo* than the other groups.

We also tested the drug release characteristics of HE/DOX. The release of HE/DOX is higher in acidic environments, which is a rational feature of cancer treatment, given that cancer cells reside in an acidic microenvironment (32, 33). We found that the release of HE/DOX in an acidic environment can reach up to 82%, whereas it was only 58% under normal physiological conditions. This increased drug release may be due to the increased solubility of DOX in acidic environments or may be attributed to the fact that the vesicle structure is more prone to disruption in acidic environments, thereby facilitating faster drug release (34). Furthermore, HE/DOX is more susceptible to protonation in acidic environments, which accelerates DOX release.

However, the mechanism underlying the targeting of osteosarcoma cells by mesenchymal stem cell-derived exosomes remains unclear. Currently, cancer targeting is primarily achieved through the receptor-ligand interactions of specific antigens on the surface of cancer cells (35). Tumor cells often overexpress numerous surface proteins compared with normal cells, which could be a probable reason for the increased internalization of HEs. In this study, using HEs prepared by the fusion of mesenchymal stem cell-derived exosomes with liposomes, we observed a significant enhancement in internalization and cytotoxic characteristics. These findings suggest promising prospects for osteosarcoma treatment.

In this study, we used BMSC-derived exosomes to modify liposome nanoparticles, providing a new method for modification of other nanoparticles. Previous studies have employed red blood cell, platelet, or leukocyte membranes to mimic cellular features and enhance the functionality of nanoparticles (36–38). Compared to cellular membrane camouflage, the exosomal membrane used in our study mimicked natural nanobiomaterials, displaying similarities in size and co-functionality, while remaining less susceptible to mononuclear phagocytic system clearance. The adhesive molecules on the exosome membrane also conferred the enhanced cell binding and internalization capabilities of HEs. Therefore, as a novel drug delivery material, HEs can effectively prolong circulation time, improve biocompatibility, and offer targeting potential.

In conclusion, our study demonstrated that mesenchymal stem cell-derived exosomes can naturally target osteosarcoma and can be utilized as a camouflage membrane to fuse with liposomes to deliver chemotherapeutic drugs for osteosarcoma treatment. Although these results are promising, we have not fully elucidated the mechanism of exosome targeting in tumors, as some studies have suggested that mesenchymal stem cell-derived exosomes can promote the progression of osteosarcoma (24). Therefore, future studies should focus on understanding the mechanisms underlying exosome targeting in osteosarcoma, such as those involving integrins, surface proteins, and lipids. Our study suggests that HEs can serve as effective carriers for chemotherapy in cancer patients.

## 4 Methods

### 4.1 Cell culture

The human BMSCs and osteosarcoma 143B and MG63 cell lines were purchased from the Peking Union Cell Bank, China. All cells were cultured in Dulbecco’s modified Eagle’s medium (DMEM) supplemented with 10% fetal bovine serum, 1% streptomycin, and 100 IU/mL penicillin. The BMSCs were cultured in exosome-free medium; specifically, the added fetal bovine serum was centrifuged at 100,000 × *g* for 16 h to remove the exosomes in the serum. All cells were cultured at 37°C in a 5% CO_2_ incubator.

### 4.2 Exosome isolation

The exosomes were isolated using ultracentrifugation. Specifically, BMSCs were cultured in DMEM without exosomes for 48 h and the cell supernatant was collected. Debris or dead cells were removed by gradient centrifugation at 300 × *g* for 10 min, at 2000 × *g* for 20 min, and at 10,000 × *g* for 30 min. The supernatant was then placed in an ultracentrifuge tube and centrifuged at 100,000 × *g* for 70 min to obtain uniform exosomes. The concentrated exosomes were resuspended in PBS and stored at −80°C for further study. All centrifugation procedures were performed at 4°C.

### 4.3 Synthesis of liposomes

The liposomes were prepared via film hydration and membrane extrusion. Specifically, L-α-phosphatidylcholine (Egg, Chicken) (EggPC) and cholesterol were uniformly mixed in chloroform at a ratio of 66:34. The mixture was dried overnight. The next day, the dried film was hydrated with PBS and fully dispersed using ultrasound. An extruder (Avanti Mini-Extruder) was used to extrude the dispersed post-solution subsequently through 400- and 200-nanometer polycarbonate membranes to obtain uniform nanoliposomes.

### 4.4 Synthesis of HEs and HE/DOX

HEs were prepared using a simple extrusion method. Specifically, the isolated exosomes and prepared liposomes were fully mixed in PBS solution at a mass ratio of 1:5 and ultrasonically treated. The mixed solution was extruded through 400- and 200-nanometer polycarbonate membranes using an extruder to obtain monodisperse HEs.

DOX was encapsulated within HEs using the ammonium sulfate concentration gradient method. Briefly, exosomes were mixed with liposomes and resuspended in 240 mM ammonium sulfate solution. After sufficient vortexing and ultrasonic treatment, HEs encapsulated in an ammonium sulfate solution were obtained through 400- and 200-nanometer polycarbonate membranes. The solution was injected into a Slide-A-Lyzer Nutritional Cassette (MWCO 20 kDa) and dialyzed overnight at room temperature in PBS solution (pH 7.4) to remove ammonium sulfate outside the HE membrane and obtain a solution with a concentration gradient. The next day, a final concentration of 0.2–0.8 mg/mL DOX was added to the HE solution and then incubated at room temperature for 6 h. Subsequently, the solution was injected into the Slide-A-Lyzer Nutritional Cassette (MWCO 20 kDa). The obtained HE solution containing DOX was named HE/DOX and stored at −80°C for subsequent research. Liposomes loaded with DOX were prepared in the same manner without exosomes and were named LP/DOX.

### 4.5 Characterization of LP/DOX and HE/DOX

The surface morphologies of LP/DOX and HE/DOX were observed using transmission electron microscopy (Hitachi, HT7700). The concentration and size distribution of LP/DOX and HE/DOX were measured using nanoparticle tracking analysis (Malvern, NanoSight NS300). The surface charges on LP/DOX and HE/DOX were characterized using dynamic light scattering (DLS, Malvern ZSP). Confocal microscopy (A1, Nikon) was used to confirm the successful encapsulation of DOX within HEs.

Positive exosomal markers (ALIX, CD63, and TSG101) and a negative marker (calnexin) in HEs and exosomes were analyzed using western blotting. RIPA lysis buffer (R0010, Solarbio) was used to extract proteins from the cells, HEs, and exosomes, and the total amount of protein was determined by BCA colorimetry. The extracted protein samples were then analyzed using polyacrylamide gel electrophoresis and western blotting. The following antibodies were used: ALIX (1:1000 dilution; Proteintech, USA), CD63 (1:1000 dilution; ABclonal Technology, China), TSG101 (1:1000 dilution; Proteintech), and calnexin (1:1000 dilution; ABclonal Technology).

### 4.6 Drug loading and release study

A standard curve was drawn using a Multimode Microplate Reader (Thermo Fisher Technologies, USA) at excitation and emission wavelengths of 480 and 594 nm, respectively, and the concentration of DOX was determined.

As mentioned above, DOX was loaded into HEs using the ammonium sulfate concentration gradient method, and the concentration of DOX added was 0.2–0.8 mg/mL (0.1, 0.2, 0.4, 0.6, and 0.8 mg/mL). The following DOX loading efficiency (LE) and encapsulation efficiency (EE) formulas were used: LE = DOX_loaded_/(HE_initial_ + DOX_loaded_) × 100% and EE = DOX_loaded_/DOX_initial_ × 100%, where DOX_loade_ is the mass of DOX loaded into HEs, HE_initial_ is the mass of HEs, and DOX_initial_ is the mass of DOX initially added to the solution.

We measured the drug release rates for HE/DOX and LP/DOX in different pH environments (pH 7.4 or 5.5). The concentrations of HE/DOX or LP/DOX were adjusted to 100 μg/mL. Next, 1 mL of HE/DOX or LP/DOX solution was added to a Slide-A Lyzer Dialysis Cassette (MWCO 20 kDa). The dialysis cassette was placed in 150 mL of PBS at pH 7.4 or 5.5. The samples were placed on a shaker at 37°C and shaken at a constant speed. At 3, 6, 12, 24, 48, and 72 h, 1 mL of the PBS dialysate was removed to determine the DOX content, and 1 mL of fresh PBS was added to replenish the sample.

### 4.7 Exosome labeling and homing to the osteosarcoma cell lines *in vitro*

Exosomes were labeled with PKH26 (Sigma-Aldrich). Specifically, 3 μL of PKH26 reagent and 500 μL of diluent C were added to 100 μg of exosome suspension and incubated at 37°C for 30 min. Free PKH26 was then removed using ultracentrifugation.

143B and MG63 cells were seeded onto 24-well culture slides at a density of 2 × 10^4^ cells/well. After 24 h of culture in an incubator, PKH26-labeled human BMSC-derived exosomes or liposomes were added. After 12 h of re-culture, all the cells were washed three times with PBS, fixed in 4% paraformaldehyde for 30 min, and then washed three times with PBS. Subsequently, 100 μL of DAPI staining solution (Beyotime, China) were added to each well for nuclear staining. Cell fluorescence was measured using an Axio Vert A1 fluorescence microscope (Carl Zeiss, Germany), and fluorescence intensity was calculated using the ImageJ software.

DiD (Thermo Fisher) was used to label exosomes. Brielfy, 10 μL of DiD reagent was added to 100 μg of exosomes and incubated at 37°C for 30 min. Free DiD was removed using ultracentrifugation. 143B and MG63 cells were seeded in 6-well plates and cultured for 24 h. DiD-labeled liposomes or BMSC-derived exosomes were added and co-cultured for 12 h. The cells were then digested with trypsin, the supernatant was removed by centrifugation, and the cells were washed twice with PBS. The cells were resuspended and analyzed using flow cytometry on a DxFLEX system (Beckman Coulter).

PKH26-labeled HE/DOX and LP/DOX were prepared following the same method.

### 4.8 Uptake of HE/DOX by tumor cells *in vitro*

Osteosarcoma 143B and MG63 cells were seeded onto glass slides in 24-well plates at a density of 2 × 10^4^ cells/well. PKH26-labeled HE/DOX were co-cultured with 143B and MG63 cells for 12 h. After washing three times with PBS, the cells were fixed with 4% paraformaldehyde for 30 min, washed three times with PBS, and stained with DAPI. Images were captured using a confocal microscope and quantitatively analyzed using the ImageJ software.

### 4.9 *In vitro* toxicity

A CCK-8 (Sigma-Aldrich) assay was conducted to analyze the toxicity of free DOXand HE/DOX in the osteosarcoma cell lines. First, 2,000 cells were seeded in 96-well plates and cultured in DMEM medium containing 10% fetal bovine serum for 24 h. The next day, the medium was replaced with fresh medium containing BMSC-derived exosomes, liposomes, HEs, free DOX, or HE/DOX and cultured for 24 h. The CCK-8 reagent was added and incubated at 37°C for 2 h. Absorbance was measured at 450 nm using a Multimode Microplate Reader (Thermo Fisher Technologies).

### 4.10 *In vivo* antitumor effect

Osteosarcoma 143B cells were inoculated into the right tibia of nude mice. After 10 days, the mice were randomly divided into four groups (*N* = 5) and injected with 150 μL of PBS, HEs, free DOX, LP/DOX, or HE/DOX every 2 d for five times. Free DOX, LP/DOX, and HE/DOX were administered at a dose of 5 mg/kg. The body weight of the mice was measured every 2 d, and the tumor volume was measured using a Vernier caliper. The formula for calculating tumor volume (*V*) is as follows:


$$\text{V}=\frac{4\prod }{3}{\left(\frac{\text{AP}+\text{L}}{4}\right)}^{2}$$

where *AP* is the distance between the tumors on both sides of the kneecap and *L* is the length of the tumor in front of the tibia.

### 4.11 Statistical analyses

All statistical analyses were performed using GraphPad Prism 8 (GraphPad software). All experimental data were described as mean ± standard deviation. Differences between groups were analyzed using two-way analysis of variance. *P* values less than 0.05 were considered statistically significant (*P < 0.05, **P < 0.01, ***P < 0.001). All the results were obtained from three replications of the experiments.

## Data Availability

The original contributions presented in the study are included in the article/supplementary material, further inquiries can be directed to the corresponding author/s.

## Supplementary material

Supplemetary Figure S1 Time-dependent doxorubicin leakage rate from LP/DOX and HE/DOX.

Supplemetary Figure S2 Cellular toxicity of exosomes, liposomes, and HE on osteosarcoma cells 143B **(A)** and MG63 **(B)** revealed that there was no significant increase in cytotoxicity with increasing concentrations.
