# Supplementary figures and images for: Bone marrow mesenchymal stem cell-derived exosomes improve cancer drug delivery in human cell lines and a mouse osteosarcoma model
Source: Front Oncol. 2024 Nov 12;14:1482087. doi: 10.3389/fonc.2024.1482087 (PMC11588629; doi:10.3389/fonc.2024.1482087)

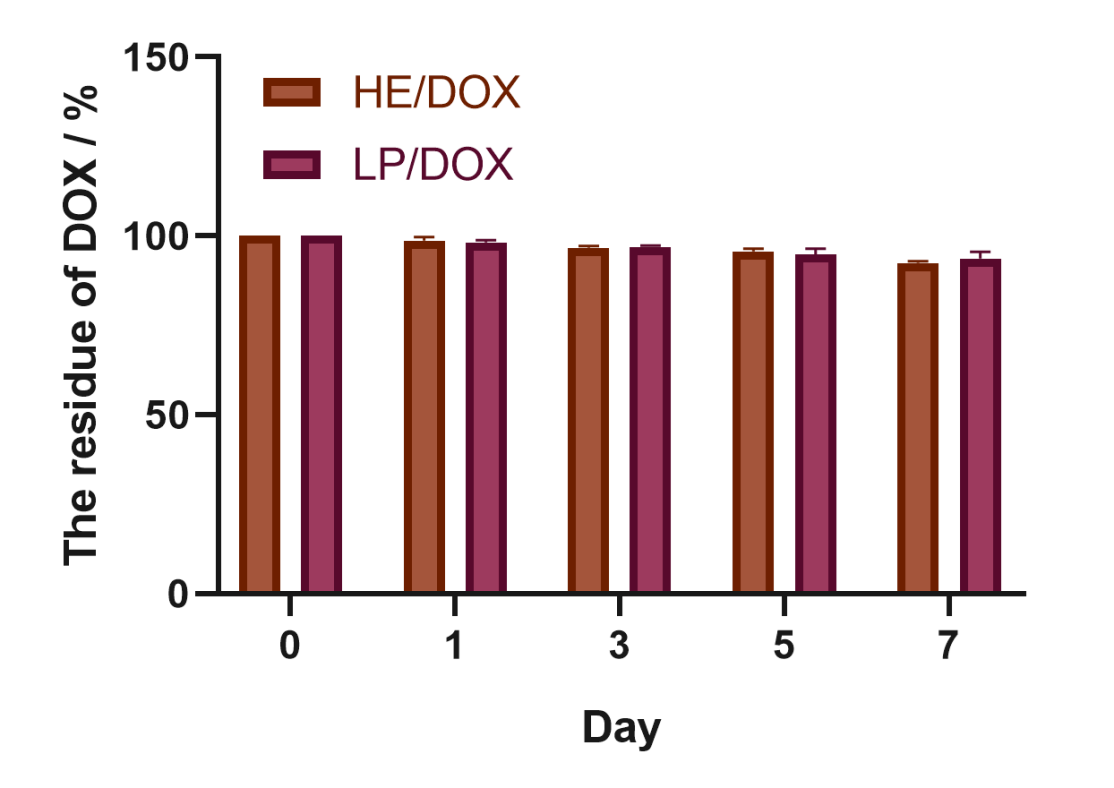

Supplement: Supplemetary Figure S1 — Time-dependent doxorubicin leakage rate from LP/DOX and HE/DOX. [file Image1.tif]

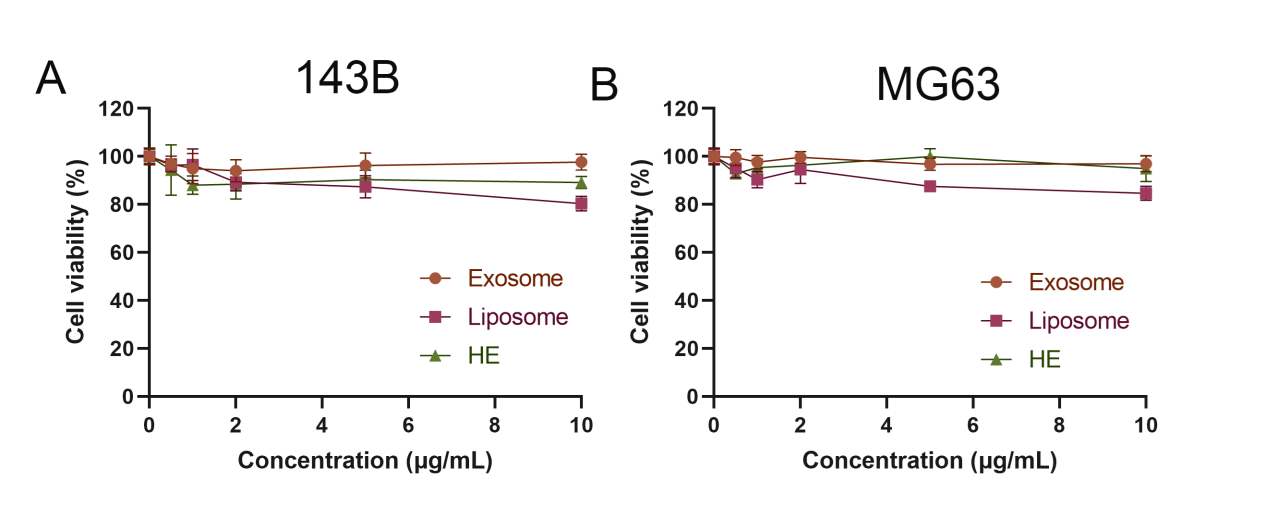

Supplement: Supplemetary Figure S2 — Cellular toxicity of exosomes, liposomes, and HE on osteosarcoma cells 143B (A) and MG63 (B) revealed that there was no significant increase in cytotoxicity with increasing concentrations. [file Image2.tif]
